# An education programme to increase general practitioners’ awareness of their patients’ employment: design of a cluster randomised controlled trial

**DOI:** 10.1186/1471-2296-15-28

**Published:** 2014-02-07

**Authors:** Kees A de Kock, Romy Steenbeek, Peter C Buijs, Peter LBJ Lucassen, J André Knottnerus, Antoine LM Lagro-Janssen

**Affiliations:** 1 Department of Primary and Community Care, Gender & Women’s Health, Radboud University Nijmegen Medical Centre, Nijmegen, PO Box 9101, 6500 HB Nijmegen, The Netherlands; 2 TNO Work and Health, Hoofddorp, The Netherlands; 3 TNO Work and Health, Rijswijk, The Netherlands; 4 Department of Primary and Community Care, Radboud University Nijmegen Medical Centre, Nijmegen, The Netherlands; 5 Department of General Practice, Maastricht University, Maastricht, The Netherlands

**Keywords:** Primary health care, RCT, General practitioners, Occupational health physicians, Gender, Absenteism, Presenteeism, Tailored training intervention, Return-to-work, Self-efficacy

## Abstract

**Background:**

Work and being able to work are important prerequisites for health and well being. Health problems can have a negative influence on the ability to work and not being able to work can be detrimental for patients’ psychosocial well being. Although GPs are aware of this importance they do not always structurally pay attention to patients’ work during their daily practice.

**Methods/design:**

To investigate whether GPs can be trained to increase their awareness of work and improve their skills when dealing with work related problems we designed a cluster randomised controlled trial. The intervention in this trial is a tailored training based on the findings of qualitative research with focus groups of GPs. Gender aspects received specific attention in these focus groups. Primary outcome measures are self efficacy of patients concerning return to work, and GPs’ use of ICPC code Z05 (work problems) and registration of patients’ occupation. Secondary outcome measures are work awareness of GPs as perceived by patients, quality of life, health, use of care and illness related costs. A process evaluation will be part of our study.

**Discussion:**

We investigate a training to increase work awareness among GPs, improve their skills in managing work related problems and structurally register work related data in the EMR. We think this study will make a contribution to better health care for workers by motivating GPs to appreciate their specific needs. It will also add to our knowledge of the complex relationship between gender, work and health.

**Trial registration:**

Trial registration number: NTR3475

## Background

Work, and being able to work, are important prerequisites for an individual’s health and well being [1]. For most people work means much more than just a source of income. Active participation in the community and making a valuable contribution lead to a higher self-esteem whereas not working often results in feelings of shame, isolation and loneliness together with their detrimental effects on health [2,3]. Although health professionals will understand the importance of their patient’s work, this is usually not reflected in the attention they pay to it in daily practice. Even general practitioners (GPs), who are trained to take into consideration the background and context of their patients, rarely pay attention to work-related health issues [4,5]. This neglect may result in patients who stay at home with minor illnesses or with chronic illnesses while both groups might be able to continue participation in their jobs after appropriate counselling by a health care professional. An important reason for the lack of attention for work-related health issues may be the fact that, unlike GPs in other countries, Dutch GPs do not have to certify sick leave [6]. This may also explain why there is a lack of research about this topic in the Netherlands and why the cooperation between GPs and occupational physicians has remained poor, despite many efforts to improve it [7].

Trying to enhance the work awareness of GPs is important, because GPs are usually the first health care professional contacted by workers with health problems, often long before these workers see an occupational physician (OP). Moreover they are generally highly trusted by the patients who explicitly prefer to be seen by their GPs during sickness absence [8]. Therefore GPs have an excellent position for early recognition and intervention of work related problems (WRP).

The lack of awareness in GPs for work problems may also be related to gender aspects. Work-related factors were found to influence the use of health care of male and female patients differently. Firstly, doctor-patient communication in general is strongly influenced by gender: male and female doctors act differently when confronted with similar problems [9,10]. Secondly, male and female patients differ in consultation behaviour. Women consult their GP more frequently and present more mental health problems [11]. Men consult less frequently, both as a result of cultural norms and practical hindrances like opening times of surgeries [12]. Men tend to identify more strongly with their jobs than women, who usually have more roles they identify with. Therefore the health consequences of losing a job can be more severe for men than for women [2]. Thirdly different factors have been shown to increase the risk for care-seeking in men and women. For men these are, for example, poor job satisfaction, routine work without opportunities for learning, physical load from bending forward and low demands in relation to competences [13,14]. For women these risk factors are, for example, reduced opportunities to acquire new knowledge, high physical loads, solitary work, and, especially for single mothers, the double burden of a job and the care of their families [2].

If work related problems are not addressed timely or properly this may contribute to long-term absenteeism from work or even to permanent disability [7]. To help medical professionals reduce the negative effects of unnecessarily lost working days, the Health Council of the Netherlands made recommendations about the management of diseases that frequently lead to long term absenteeism [15]. In its professional “Core Values” The Dutch College of General Practitioners recently addressed the importance of proactively paying attention to work [16]. Unfortunately this is not reflected yet in most of their guidelines [17].

There is limited evidence that GPs can be trained to improve their performance regarding sickness certification. Cohen et al. found an increased doctor reported self-efficacy among GPs in the UK after a tailored training [18,19]. Østerås et al. found that implementation of a structured functional assessment by Norwegian GPs influenced their prescription of part time and full time sick leave but had no effect on the duration of sick leave episodes [20]. Van Dijk et al. showed that the registration of risk factors for long-term sickness absence by Dutch GPs could be improved by using a protocol [21].

We conclude that there is a lack of attention of GPs to work-related health problems and that gender differences may play a role. We assume that education of GPs can increase their awareness of the possible two way relation - either causal or conditional - between work and presented health problems. This may lead to a better understanding of work-related problems and improve treatment strategies with benefits for both individual patients and society as a whole. Therefore we developed a GP education programme to increase GP awareness of work, also addressing the influence of gender of patients and GPs. The aim of this paper is to describe the design of a cluster randomised controlled trial about the cost-effectiveness of this programme. We will also evaluate the process of implementation.

## Methods

### Trial design

The study is a cluster randomised controlled trial using randomisation on practice level to prevent contamination. We will randomise the practices into two groups with a 1:1 allocation ratio. GPs in the intervention group practices will receive the training program, GPs in the control practices will deliver care as usual.

### Participants

#### General practitioners

GPs working in the South-Eastern part of the Netherlands will be asked by letter to participate in the study. If an insufficient number of GPs respond positively to the letter, GPs will be approached more directly by email and telephone. Only GPs who work at least 2 days per week will be recruited.

#### Patients

The patient population will exist of patients (from the lists of the participating GPs) who are 18–63 years of age, have paid work (paid-employment or self-employed) for at least 12 hours per week and have sufficient understanding of the Dutch language to fill in a questionnaire. The upper age limit of 63 was chosen to lower the chance that patients drop out during follow-up due to retirement.

### Randomisation

We aim to recruit at least 24 GPs who will be randomised into an intervention group and a control group. To prevent contamination, we will randomise clusters on practice level. A statistician will generate a block randomisation scheme using a block size of 2 and practices will be assigned to either condition according to the order in which they are recruited. After this order is established the allocation of the random sequence will be performed by the researcher.

### Intervention

We developed our intervention based on the literature and on the results of four focus groups with field experts of male and female GPs. This showed the importance of an active role of the GP [Qualitative article in progress]. Apart from lack of knowledge, for instance about legislation and gender, concern to become involved in a conflict between patient and employer turned out to be an important hindrance for GPs to address work. Therefore, the training has to stress the benefits of a proactive policy which entails coaching the patient to remain (or get) on speaking terms with the other relevant actors, thereby preventing or resolving conflicts. An accredited five hour training-intervention was developed to help GPs adjust their working style accordingly, covering the following items.

1. The connection between work and health (lecture).

2. Possibilities to improve usual care (discussion participants).

3. The rules regarding work and absenteeism and ways to come to a fruitful collaboration between GPs and OPs (lecture).

4. The gender aspects of work and work related problems (lecture).

5. Recommendations regarding a pro-active approach (lecture) and workshops about how to bring these recommendations into practice.

6. Advice regarding registration of work-related data in the electronic medical files (lecture)

7. Instructions regarding the data collection for the trial.

Two months after the initial training the participants will be offered a three hour booster training. Cases of the participants will be discussed with the researcher and the OP during the first part of these sessions. During the second part, consensus will be sought about feasible ways for GPs to structurally register work related data. These booster training sessions will be offered on two different days to optimise participation by intervention group GPs.

### Procedures

Inclusion will take place during a 4 month period, starting after the training of the intervention group GPs. The GPs’ receptionists will approach patients in the waiting room with a short questionnaire about age, number of working hours and their willingness to participate in the project. If a patient meets the selection criteria and signs informed consent the patient will be included and receive the first questionnaire. A second and third questionnaire will follow after 6 and 12 months. Forty patients for each participating GP, 20 females and 20 males, will be included for the study.

Only the intervention group GPs will be asked to fill in a form for each consenting patient with questions about: 1 the occupation of the patient; 2 the work relatedness of the problem that he/she presented; 3 whether sick leave was discussed; 4 whether the GP had suggested contacting an OP; 5 whether the GP had stimulated the patient to adopt a pro-active coping style. Two final questions asked whether the GP thought the training had been helpful for the consultation and if the GP had enough time for the consultation.

The control group will deliver care as usual. As it could be surmised that their usual care is influenced by the data collection we try to make sure that the data collection in the control group practices is carried out as much as possible by the receptionists supported by a research assistant. When data collection is completed the control group GPs are offered the same training as the intervention group as an incentive for adherence to the study protocol.

Patients will be approached by the receptionist before the consultation and, if they are prepared to participate in the study, are asked to answer the first questionnaire after the consultation and a second and third questionnaire after six and twelve months respectively.

### Outcome measures

#### Primary outcome measures

As primary outcome measures at GP level we use the registration of patients’ employment in the electronic medical record (EMR) and the use GPs make of the code Z05 (for work related problems) of the International Classification of Primary Care (ICPC). For each participating GP we establish the percentage of the working age patients for whom the occupation is recorded anywhere in the EMR and we count the instances in which they made use of code Z05. The registration of employment and use of code Z05 in the 6 months following the training will be compared between intervention and control group practices.

The primary outcome measure at patient level is the work-related self-efficacy, measured by the 11 item Return-to-Work Self-Efficacy scale ( RTW-SE) [22]. This scale measures the extent to which people feel able to handle the demands of their job on a scale which ranges from 1 to 6. The RTW-SE is validated and reliable and has a high internal consistency. It was found to strongly correlate to return to work in a sample of sick listed workers; it was also shown to be sensitive to changes in the clinical condition of patients suffering from mental health problems [23].

Outcome measures regarding gender are the use of code Z05 and the registration of occupation in women and men. Moreover we will analyse the score on the RTW-SE scale and investigate its relation to gender and how this is modified by other patient variables like age, occupation, income, health problems, absenteeism and other patient variables.

#### Secondary outcome measures

The secondary outcome measure at GP level is GP’s attention for the patients’ work related problems as it is perceived by patients. It is measured by a self-constructed five item scale, with 2 response categories: “yes” or “no” concerning: 1. Does the GP know your occupation? 2. Do you think the problem for which you consulted the GP is work related? 3. Was this possible relationship discussed by your GP? 4. Did your GP discuss sick leave? 5. Did the GP help you to find solutions for work-related problems?

As mentioned above, intervention group GPs are asked to fill in a form for each participating patient with questions on the same topics. This will provide us with information about the extent to which intervention group GPs and patients agree on these issues.

Secondary outcome measures at patient level consist of productivity, sick leave duration, health related to work, quality of life, self-rated health and direct illness-related costs, health care and non-health care. Measures for productivity are both absenteeism and presenteeism. Absenteeism refers to the total days lost from work. Presenteeism refers to attending work whilst still sick or disabled, causing reduced productivity while at work [22,24,25]. Sick leave and presenteeism will be measured with modules C and D of the Productivity and Disease Questionnaire (Prodisq) [26]. The Prodisq is developed based on the Quantity and Quality (QQ) method and provides a reliable and valid tool for measuring quantity and quality of work on a daily basis [25,26]. Quality of life will be measured by means of the RAND-36 and EQ-5D-5 L [27,28]. The RAND-36 consists of 8 subscales: physical functioning, role limitations due to physical problems, social functioning, bodily pain, general mental health, role limitations due to emotional problems, vitality, and general health perceptions [28]. The EQ-5D-5 L is an improved version of the Euroqol (EQ-5D). In contrast to the Euroqol, the EQ-5D-5 L has 5 levels of severity in 5 dimensions, instead of 3 levels of severity in 5 dimensions, which improves the instrument’s sensitivity [28].

Self-rated health will be assessed using one item from the Short Form Health Survey (SF-36). This widely used measurement tool describes how a respondent values and assesses his/her general health [29]. To detect health complaints and co-morbidity underlying work disability, questions derived from The Netherlands Working Conditions Survey (NWCS) will be used [30].

Direct costs, both healthcare and non-healthcare, will be measured by an adapted version of the Trimbos/iMTA questionnaire (TiC-P) [31].

### Process evaluation

A short questionnaire and individual interviews will be used to examine whether there are any identifiable facilitating factors and perceived barriers for implementation of our education programme.

### Cost-effectiveness evaluation

The cost-effectiveness of the intervention will be evaluated from the perspective of society at large whereby the costs and benefits will be captured independently of those who bear the costs and those who receive the benefits. We will measure and value health care costs and non health care costs, like productivity costs and costs related to the intervention. Health care costs include costs directly related to the provision of health care, such as costs for primary and secondary care, but also costs for drugs and alternative treatments. These costs will be calculated following the cost calculation guidelines for health care in the Netherlands [32]. The prices of prescribed drugs will be based on Daily Defined Dosage (DDD) taken from the Royal Dutch Society for Pharmacy [32]. The direct non-health care costs are calculated using the information obtained from the cost questionnaires and shadow prices. Productivity costs are costs in paid labour as a consequence of sickness, sick leave and disability of a productive person. The most important productivity costs will be measured in terms of lost productivity: presenteeism, absenteeism and compensation mechanisms. Lost productivity will be calculated by using the friction cost method which basically multiplies the days of production loss until replacement by the average day wage [32]. For the latter method, the Dutch guideline for economic evaluation is used [32]. The costs directly related to the development and implementation of the intervention will be registered. Detailed information concerning the methods of economic evaluation can be found in the article by Noben et al. [33].

### Statistical analysis

#### Sample size

The sample size calculation was based on the outcomes of the RTW-SE in a reference group consisting of employees who were sick listed for more than 13 weeks. Here the mean score was 4.24 with a standard deviation of 1.14. We want our study to allow us to demonstrate a moderate effect of our intervention. Cohen defined a moderate effect as a difference of a half standard deviation [34]. Therefore we need two groups of at least 12 GPs, based on a power of 80% and an alpha of 0,05. Assuming a loss of 25%, they have to recruit 40 patients each to be able to analyse 30 patients. This calculation is based on a conservative estimate of the intra cluster correlation coefficient of 0.15.

#### Effect evaluation

The outcomes of the questionnaires will be compared between both groups at the three different measurements. Descriptive statistics will be used to summarise the data and detect outliers, missing values and data entry mistakes. We will describe the categorical variables using frequency tables; for each continuous variable we will calculate minimum, maximum, range, median, mean and standard deviation. The data will be further analysed using a multi level model to assess the influence of the different variables. All analyses will be performed at patient level according to the intention to treat principle, so patients will be regarded as belonging to the intervention group if their GP was allocated to the intervention condition, regardless of whether the GP effectively received the training.

The primary independent variable in the analyses will be the GPs’ exposure to the training: trained GP or control group delivering usual care. The primary dependent variables are their patients’ score on the RTW-SE scale, the number of Z05 codes used by the GPs and the proportion of working age patients for whom the occupation is registered. Effects of the intervention will be checked for effect modification by experience and gender of the GPs. All analyses will be performed with SPSS. Effects on the different outcome variables will be assessed at a significance level of 0.05.

### Ethical approval

The institutional ethics review board was consulted and concluded that approval was not needed according to the Dutch law (letter CMO 6th April 2011). Patients will be asked to sign informed consent.

## Discussion

In this study, we will investigate whether GPs can be trained to more consistently pay attention to work aspects of health problems and to adopt a pro-active consultation style. We will also investigate whether this increases work-related self-efficacy in their patients. At this moment health problems that are related to work often linger on for a long time and can result in presenteeism and absenteeism without them being addressed properly by health professionals. We hypothesize that if GPs broaden their scope to include the working environment of their patients, more problems will be recognized in a phase in which it is easier to find solutions.

### Strengths and weaknesses of the study

An important strength of our study is the tailored character of the intervention that is based on the findings of our own focus group research. The training is expected to change the behaviour of GPs by making them more aware of the relationship between work, health and gender. This will enable them to recognize WRP in an earlier stage and this will also help them to use more effective therapeutic strategies.

In many trials regarding work related health problems, interventions aim at reducing sick leave. However a risk of taking sick leave as an outcome measure lies in it defining sick leave as the main problem rather than the conditions and circumstances that it is a result of. By our focus on work per se, including its important positive effects on health and well being, we think we can help GPs to better appreciate the meaning of work for patients. Using the beneficial effects of participation may be better suited to the preferences of GPs and the position of trust they have with their patients than playing a role in sickness certification. Once they acknowledge the important positive influence they can exert in this field for their patients, GPs may be motivated to further take up this task.

Another strength lies in the outcome measures we use. Currently, the registration by GPs of patients’ occupations and work related problems is inadequate. Even motivated GPs often do not know the relevant ICPC codes. This leaves much room for improvement making it more likely to find a significant effect of our intervention. Such an effect will also be relevant as registration of work related problems unequivocally shows that GPs pay attention to work. Improving the registration in the EMR has been subject of a government policy. Incentives are used to motivate GPs for adequate ICPC coding in the EMR. This may further stimulate GPs to use the ICPC codes for work related problems. The use of the RTW-SE scale in a primary health care setting will shed new light on the work-health relation of workers visiting their GP. Even if we do not find any effect on this scale, our data may be invaluable in further developing knowledge about the relationship between return-to-work self efficacy, absenteeism and presenteeism. Moreover, it will offer us insight in the possibilities of this instrument in a primary care setting, for instance as a diagnostic tool.

A weakness at patient level is the fact that most of the data are collected by self-reported questionnaires. This method is always subject to recall bias. However, since both the intervention- and control group will experience this bias, we do not expect that it will influence the comparison between the groups. Another weakness concerns the selection of patients. Only patients who are willing to participate are included This means our findings may not be transferable to all working patients. This effect is expected to occur in comparable ways in both groups.

A weakness at the GP level is the selection of GPs who are willing to participate. We assume that these GPs will be more open to new ideas and more positive concerning work-related issues. This may result in a smaller effect as there will be less room for improvement in the selected group. On the other hand, implementation of the programme amongst GPs in general is assumed to lead to a smaller effect. Heterogeneity of the group of GPs may be another problem. As work gets little attention during the vocational training and in quality improvement schemes of GPs, no ‘standard’ way of dealing with it has evolved. Therefore we expect to find a considerable inter-GP variation which, in its turn, can make it harder to reliably assess the effect of the training intervention.

We think that our study can mean an important step forward in health professionals paying more attention to work. This can lead to more effective and efficient health care for workers.

## Competing interests

The authors declare that they have no competing interests.

## Authors’ contributions

All authors contributed to the design of the study and the writing of this paper. KdK is the principle researcher and responsible for the data collection. All authors read and approved the final manuscript.

## Pre-publication history

The pre-publication history for this paper can be accessed here:

http://www.biomedcentral.com/1471-2296/15/28/prepub
